# Aging effect of cross-modal interactions during audiovisual detection and discrimination by behavior and ERPs

**DOI:** 10.3389/fnagi.2023.1151652

**Published:** 2023-04-26

**Authors:** Yanna Ren, Yan Li, Zhihan Xu, Rui Luo, Runqi Qian, Jieping Duan, Jiajia Yang, Weiping Yang

**Affiliations:** ^1^Department of Psychology, College of Humanities and Management, Guizhou University of Traditional Chinese Medicine, Guiyang, China; ^2^Department of Foreign Language, Ningbo University of Technology, Ningbo, China; ^3^Applied Brain Science Lab Interdisciplinary Science and Engineering in Health Systems, Okayama University, Okayama, Japan; ^4^Department of Psychology, Faculty of Education, Hubei University, Wuhan, China

**Keywords:** aging, audiovisual integration (AVI), ERP, race model, older adults

## Abstract

**Introduction:**

Numerous studies have shown that aging greatly affects audiovisual integration; however, it is still unclear when the aging effect occurs, and its neural mechanism has yet to be fully elucidated.

**Methods:**

We assessed the audiovisual integration (AVI) of older (*n* = 40) and younger (*n* = 45) adults using simple meaningless stimulus detection and discrimination tasks. The results showed that the response was significantly faster and more accurate for younger adults than for older adults in both the detection and discrimination tasks. The AVI was comparable for older and younger adults during stimulus detection (9.37% vs. 9.43%); however, the AVI was lower for older than for younger adults during stimulus discrimination (9.48% vs. 13.08%) behaviorally. The electroencephalography (EEG) analysis showed that comparable AVI amplitude was found at 220–240 ms for both groups during stimulus detection and discrimination, but there was no significant difference between brain regions for older adults but a higher AVI amplitude in the right posterior for younger adults. Additionally, a significant AVI was found for younger adults in 290–310 ms but was absent for older adults during stimulus discrimination. Furthermore, significant AVI was found in the left anterior and right anterior at 290–310 ms for older adults but in the central, right posterior and left posterior for younger adults.

**Discussion:**

These results suggested that the aging effect of AVI occurred in multiple stages, but the attenuated AVI mainly occurred in the later discriminating stage attributed to attention deficit.

## 1. Introduction

Vision and audition are two important sense organs, and the merging of visual information and auditory information can occur automatically, which is called audiovisual integration (AVI; Stein and Meredith, 1993; Scheliga et al., 2022). Integration of available auditory and visual information from the complex outside environment assists individuals in accurately perceiving the outside world, and numerous studies have found that the response to multisensory audiovisual information is faster and more accurate than that to uni-sensory auditory information or visual information across the lifespan (Brandwein et al., 2011; Ren et al., 2020c). Aging is a major global issue, and the proportion of the elderly population is increasing yearly. Aging is associated with declines in various functions, including vision, audition, tactile, olfaction and gustation (Isaev et al., 2019), and older adults are more dependent on information merging from different sensory modalities (Grady, 2012; Freiherr et al., 2013). Therefore, how elderly individuals integrate valuable information from visual and auditory sensory modalities has become a hot topic of age-related cognition, which is a key factor in the development of cognitive interventions (Ren et al., 2020c).

Using meaningful semantic stimuli, including visual colorful circles and auditory color naming, Laurienti et al. (2006) first reported that the AVI was enhanced for older adults compared with younger adults in behavior (Laurienti et al., 2006). According to their results, they proposed an assumption that older adults might establish compensatory mechanisms during multisensory audiovisual processing to ease uni-sensory functional decline. To clarify the neural mechanism for the enhanced AVI, Diaconescu et al. investigated the aging effect of AVI during processing meaningful semantic audiovisual stimuli using MEG (Diaconescu et al., 2013), and they found posterior parietal and medial prefrontal activity in charge of the age-related AVI. Specifically, preferential activity in posterior parietal and medial prefrontal regions responded to multisensory audiovisual stimuli between 150 and 300 ms, and increased activity in inferior parietal and medial prefrontal regions 100 ms after stimulus onset in older adults only. In the aforementioned studies, semantic stimulus material was applied, which induced perceptual AVI processing and semantic processing. It is difficult to disentangle the aging effect on the AVI or high-level semantic processing. In addition, semantic meaning (Doehrmann and Naumer, 2008; Ren et al., 2020b) and task complexity (Pronina et al., 2022) greatly modulate AVI processing, and investigation of the AVI of meaningless stimulus material is necessary to uncover the aging effect of AVI.

Peiffer et al. (2007) and Ren et al. (2020a,b,c) investigated the aging effect of AVI using meaningless stimulus materials behaviorally to eliminate most high-order cognitive processing, but conflicting results were obtained (Peiffer et al., 2007; Ren et al., 2020b). In the study by Peiffer et al., the visual stimulus was green light emitting diodes and the auditory stimulus was white noise, and a simple detection task required participants to respond when detecting any auditory and visual signal was conducted (Peiffer et al., 2007). They reported a higher AVI for older adults than for younger adults, consistent with Laurienti et al. (2006). However, Ren et al. designed a discrimination task that instructed participants to identify target signals (white-black checkerboard contained two black dots in white board and white noise) from nontarget signals (white-black checkboard and pure tone), and reduced the AVI for older adults compared with younger adults (Ren et al., 2020b). The detection of information in the external world is the most basic ingredient of perception because it merely contains a behavioral judgment about the presence or absence of something regardless of its identity or properties that are necessary for discrimination tasks (Pennartz, 2015). Compared with the detection task, the discrimination task requires higher cognitive processing and is also the basic ingredient of perception (Spotorno et al., 2016). Considering the importance of detection and discrimination in human life, it is necessary to clarify whether the aging effect mainly occurred at the detection level or in a relatively higher discrimination process.

Although there is a mass of recent studies concerning age-related AVI, they mainly focused on the interaction between AVI with attention or spatiotemporal synchronism (Wang et al., 2017; Ren et al., 2018, 2020a, 2022; Wang et al., 2018), and they found that compared with younger adults, additional brain networks were recruited and higher brain functional connectivity was evoked during AVI for older adults. It remains unclear when the aging effect occurs during AVI. In the current study, older and younger adults were instructed to perform meaningless auditory and visual signal detection tasks and discrimination tasks during EEG recording. This allowed us to answer two overarching research questions. First, when does the aging effect begin to influence AVI? Second, what is the neural mechanism underlying the aging effect on AVI?

## 2. Methods

### 2.1. Participants

Forty-five older adults and 45 younger adults were recruited to participate in the study. All participants were paid for their time, and 40 older adults (55–75 years old, mean age ± SD, 58.9 ± 4.4) and 45 younger adults (18–23 years old, mean age ± SD, 19.9 ± 1.1) completed the experiment successfully. Three of the older adults were unable to complete the discrimination task, and the accuracy of two older adults was lower than 60%; therefore, the data of the five older adults were excluded from further analysis. All of the older adults were recruited from Guiyang City, and all of the younger adults were college students and graduate students of Guizhou University of Traditional Chinese Medicine. The participant who takes drugs related to mental illness was excluded. All participants had normal hearing and normal or corrected-to-normal vision and were naive about the purpose of the experiment. Vision was examined by a Chinese Eye Chart, and audition was examined by Pure-tone Audiometry. The mini-mental state examination (MMSE) scores and Montreal cognitive assessment (MoCA) scores were greater than or equal to 26 (Bravo and Hébert, 1997; Jia et al., 2021). Additionally, all participants provided written informed consent before the experiment, which was previously approved by the Second Affiliated Hospital of Guizhou University of Traditional Chinese Medicine.

### 2.2. Stimuli and procedure

#### 2.2.1. Detection task

**Figure 1:**
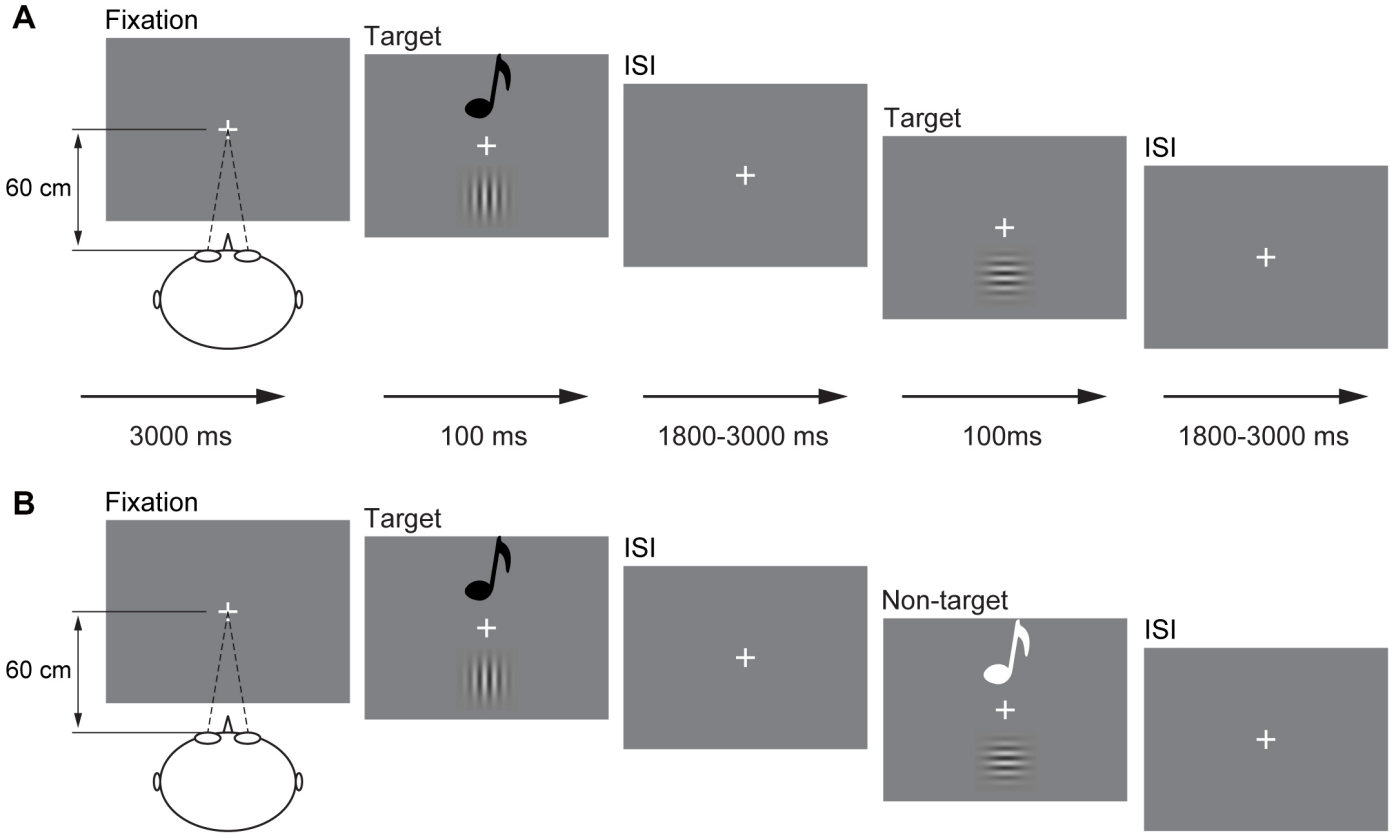
Schematic depiction of the experimental design. An example of a possible sequence of audiovisual stimuli and visual stimuli in the detection task **(A)** and a possible sequence of audiovisual stimuli in the discrimination task **(B)**. ISI, interstimulus interval.

The visual stimulus (V) is 10% contrast 1.5 spatial frequency Gabor, including horizontal Gabor and vertical Gabor. The auditory stimulus (A) is a sinusoidal tone, including 1,000 and 500 Hz. The audiovisual stimulus (AV) is the combination of 10% contrast 1.5 spatial frequency vertical Gabor and 1,000 Hz sinusoidal tone and of 10% contrast 1.5 spatial frequency horizontal Gabor and 500 Hz sinusoidal tone. No other combination of A stimulus and V stimulus was used in the current study. Participants were instructed to perform the experiment in a dimly lit, electrically shielded and sound-attenuated room (laboratory room, Guizhou University of Traditional Chinese Medicine, China). All V stimuli were presented on the center of the monitor with a gray background (RGB: 192, 192, and 192) in front of the participant (60 cm), and A stimuli were presented through speakers located centrally on the back of the monitor at 60 dB (10 ms of rise or fall cosine gate). The experiment began with a fixation “+” at the center of the screen for 3,000 ms (Figure 1A). Then, the A, V, and AV stimuli were presented randomly for 100 ms with a random interstimulus interval (ISI) of 1,800–3,000 ms. The participants were instructed to press the left button of the mouse to respond to all stimuli they perceived as rapidly and as accurately as possible.

#### 2.2.2. Discrimination task

The presentation of A, V, and AV stimuli was the same as that in the detection task but in a different response mode. In the stimulus discrimination task, the participant was only instructed to respond to vertical Gabor, 1,000 Hz sinusoidal tone, and combination of vertical Gabor and 1,000 Hz sinusoidal tone (target); however, the stimulus was withheld to horizontal Gabor, 500 Hz sinusoidal tone, and the combination of horizontal Gabor and 500 Hz sinusoidal tone (non-target; Figure 1B). The sequence of the detection task and discrimination task was random for each participant. There are 300 trials for each task, including the A, V, and AV target trials 20 times each and the A, V, and AV non-target trials 80 times each. Each task lasted for 12 min, which divided into two sessions with self-time break.

### 2.3. Data collection

The stimuli presentation and behavioral data collection were controlled using E-prime 3.0 software (Psychology Software Tolls, Inc., Pittsburgh, PA, USA). The EEG signals were recorded using the BrainVision actiCHamp Plus system (Brain Products GmbH, Gilching, Germany) through 32 Ag/AgCl electrodes mounted on an electrode cap (actiCAP GmbH, Herrsching, Germany). The vertical eye movements and eye blinks were measured by acquiring EOG data from an electrode placed approximately 1 cm below the subject’s left eye (VEOG), and the horizontal eye movements were measured by acquiring the EOG signal from one electrode placed approximately 1 cm from the outer canthi of the left eye (HEOG). The reference electrode was Fz, and the impedance was maintained below 5 kΩ. The raw signals were digitized using a sample frequency of 1,000 Hz, and all data were stored digitally for off-line analysis.

### 2.4. Data analysis

#### 2.4.1. Behavioral data

##### 2.4.1.1. Hit rate and response time

The hit rate is the percentage of correct responses (the response time falls within the average time period ±2.5 SD) relative to the total number of target stimuli. The hit rates and response times (RTs) were computed separately for each participant and then submitted to a 2 (group: older, younger) × 3 (stimulus type: A, V, AV) analysis of variance (ANOVA) (Greenhouse–Geisser corrections with corrected degrees of freedom). The statistical analysis was conducted using IBM SPSS statistic 22.0 (IBM Corp., Armonk, NY, USA), the statistical significance level was set at *p* ≤ 0.05, and the effect size (η*_P_*^2^) estimates were reported.

##### 2.4.1.2. Race model

As in our previous study on the interaction between attentional load and AVI (Ren et al., 2021, 2023), the occurrence of AVI was assessed using a race model by cumulative distribution functions (CDFs; Miller, 1982, 1986). *P*_A_, *P*_V_, and *P*_AV_ are the probability of responding within a given timeframe in a unimodal visual trial, unimodal auditory trial, and bimodal audiovisual trial, respectively. The race model (*P*_RM_) is a statistical prediction model [*P*_RM_ = (*P*_A_ + *P*_V_) − *P*_A_ × *P*_V_], and Miller (1982, 1986) proposed that *P*_AV_ will never exceeds P_RM_. If P_RM_ is significantly greater than *P*_AV_, the interaction between auditory stimulus and visual stimulus is considered to occur. To assess the amount of AVI in various conditions, a difference probability curve was generated by subtracting a subject’s race model CDF from his or her AV CDF in each 10-ms bin (Laurienti et al., 2004, 2006; Peiffer et al., 2007; Hugenschmidt et al., 2009). The peak of the difference probability curve (peak benefit) was computed separately for each participant in each condition to assess the amount of AVI. The time point of peak benefit was defined as the peak latency, and the time interval at which a significant difference occurred between the AV CDF and the race model CDFs was defined as the time window of AVI, which was used to assess when the AVI occurred together with peak latency.

#### 2.4.2. EEG data

The EEG data were imported and processed with MATLAB R2013b (MathWorks, Inc., Natick, MA, United States) with the freely available EEGLAB toolboxes^1^ (Swartz Center for Computational Neuroscience, La Jolla, CA, United States). The EEG data were positioned according to the 32-channel montage of the international 10/20 system, and only the EEG signals elicited by vertical Gabor, 1,000 Hz sinusoidal tone, and combination of vertical Gabor and 1,000 Hz sinusoidal tone were analyzed. The two electrodes monitoring eye movement (HEOG and VEOG) were deleted, and then, the data were rereferenced to the bilateral mastoid electrodes (TP9 and TP10). The original reference data were recovered to Fz. The remaining continuous EEG data were bandpass filtered from 1 to 40 Hz during recordings at a sampling rate of 1,000 Hz. For the detection task, the data were divided into epochs with 400 time points (100 ms prestimulus and 300 ms poststimulus points) and 700 time points (100 ms prestimulus and 700 ms poststimulus points) for the discrimination task. Then, an independent component analysis (ICA) was used to remove artifacts from the data, including eye artifacts, frequency interference, muscle artifacts, head movement, and electrocardiographic activity (Makeig et al., 1997; Jung et al., 2001; Delorme and Makeig, 2004). Subsequently, baseline corrections were made based on the 100 ms to 0 ms prestimulus interval data from the ICA-corrected data. The data were then averaged for each stimulus type, following digital filtering with a bandpass filter of 0.01–40 Hz, and the grand-averaged data were obtained across all participants for each stimulus type in each electrode. The AVI was calculated according to the previous studies, which have reported that audiovisual integration could be assessed by the difference in amplitude [ERP_AV_ – ERP_(A + V)_] between the sum of the event related potential (ERP) waves of the unimodal visual and unimodal auditory stimuli ERP_(A + V)_ and the ERP waves of the bimodal stimuli ERP_AV_ (Giard and Peronnet, 1999; Talsma et al., 2007).

According to previous studies (Diaconescu et al., 2013; Yang et al., 2022), five regions of interest (ROIs) were selected: left anterior (F3, FC5, and FC1), right anterior (F4, FC6, and FC2), central (C3, Cz, and C4), left posterior (P3, CP5, and CP1), and right posterior (P4, CP6, and CP2). To measure the AVI diversity between groups in each task, statistical analysis was conducted in the following steps. First, pointwise running *t*-tests between ERP_(A + V)_ and ERP_AV_ were applied. If 20 or more consecutive points were significant (20 points = 20 ms, criterion *p* < 0.050), we defined that the AVI occurred (Guthrie and Buchwald, 1991; Senkowski et al., 2007). Second, in each ROI, the amplitudes of [ERP_AV_ – ERP_(A + V)_] across each significant time interval were averaged. Finally, the mean amplitudes were submitted to 2 (group: older, younger) × 5 (ROIs: left anterior, right anterior, central, left posterior right posterior) ANOVA (Greenhouse–Geisser corrections with corrected degrees of freedom). The statistical analysis was conducted using IBM SPSS statistic 22.0 (IBM Corp., Armonk, NY, USA), the statistical significance level was set at *p* ≤ 0.05, and the effect size (η*_P_*^2^) estimates were reported.

## 3. Results

### 3.1. Hit rate and response time

#### 3.1.1. Detection task

The mean hit rate and RTs are shown in Table 1. A 2 (group: older, younger) × 3 (stimulus type: A, V, AV) ANOVA of hit rate found a significant main effect of group [*F*(1, 83) = 6.632, *p* = 0.012, η*_P_*^2^ = 0.074], indicating a higher hit rate for younger adults than for older adults, and a main effect of stimulus type [*F*(2, 166) = 32.039, *p* < 0.001, η*_P_*^2^ = 0.279], indicating a higher hit rate to the AV stimulus than to the V stimulus and A stimulus (AV > A > V, all *ps* ≤ 0.006). There was no significant interaction between group and stimulus type [*F*(2, 166) = 2.200, *p* = 0.121, η*_P_*^2^ = 0.026]. ANOVA of RTs revealed that there was a main effect of group [*F*(1, 83) = 193.620, *p* < 0.001, η*_P_*^2^ = 0.700], indicating a faster response for younger adults than for older adults. In addition, the main effect of stimulus type was also significant [*F*(2, 166) = 89.247, *p* < 0.001, η*_P_*^2^ = 0.455], indicating a faster response to the AV stimulus than to the A stimulus and V stimulus (V vs. A, *p* = 0.159). However, no significant interaction between group and stimulus type was found [F(2, 166) = 0.444, *p* = 0.587, η*_P_*^2^ = 0.005].

**Table 1:** The mean response time and hit rate with standard deviation (mean ± SD) for visual, auditory and audiovisual stimuli in the detection task and discrimination tasks.

	Detection task			Discrimination task		
	V	A	AV	V	A	AV
**Response time (ms)**						
Older	472 ± 91	504 ± 147	407 ± 107	615 ± 67	636 ± 106	550 ± 81
Younger	396 ± 60	410 ± 96	338 ± 69	554 ± 63	544 ± 80	457 ± 64
**Hit rate (%)**						
Older	88 ± 8	92 ± 8	96 ± 3	92 ± 9	92 ± 8	97 ± 4
Younger	93 ± 4	94 ± 5	96 ± 2	93 ± 6	95 ± 4	97 ± 2

A, auditory stimulus; V, visual stimulus; AV, audiovisual stimulus.

#### 3.1.2. Discrimination task

A 2 (group: older, younger) × 3 (stimulus type: A, V, AV) ANOVA revealed that there was a significant main effect of stimulus type on hit rate [*F*(2, 166) = 16.483, *p* < 0.001, η*_P_*^2^ = 0.166], indicating a higher hit rate for the AV stimulus than for the V stimulus and A stimulus (V vs. A, *p* = 0.211). No significant main effect of group [F(1, 83) = 2.753, *p* = 0.201, η*_P_*^2^ = 0.032] or interaction between group and stimulus type [*F*(2, 166) = 0.671, *p* = 0.483, η*_P_*^2^ = 0.008] was found. ANOVA also revealed that there was a main effect of group on RT [*F*(1, 83) = 32.483, *p* < 0.001, η*_P_*^2^ = 0.281], indicating a faster response in younger adults than in older adults, and a significant main effect of stimulus type on RT [*F*(2, 166) = 87.855, *p* < 0.001, η*_P_*^2^ = 0.514], indicating a faster response to the AV stimulus than to the V stimulus and A stimulus (V vs. A, *p* = 0.999). Additionally, the interaction between group and stimulus type was also found to have a significant effect on RT [*F*(2, 166) = 3.735, *p* = 0.036, η*_P_*^2^ = 0.043]. Further *post hoc* analysis was applied. The pairwise comparison for group found that the response of younger adults was faster than that of older adults to all stimuli. The pairwise comparison for stimulus found that the response to the AV stimulus was faster than to the A stimulus and V stimulus for both younger and older adults (all *p* < 0.001), but no significant difference was found between the V stimulus and A stimulus for both older (*p* = 0.203) and younger (*p* = 0.999) adults.

### 3.2. Race model

**Figure 2:**
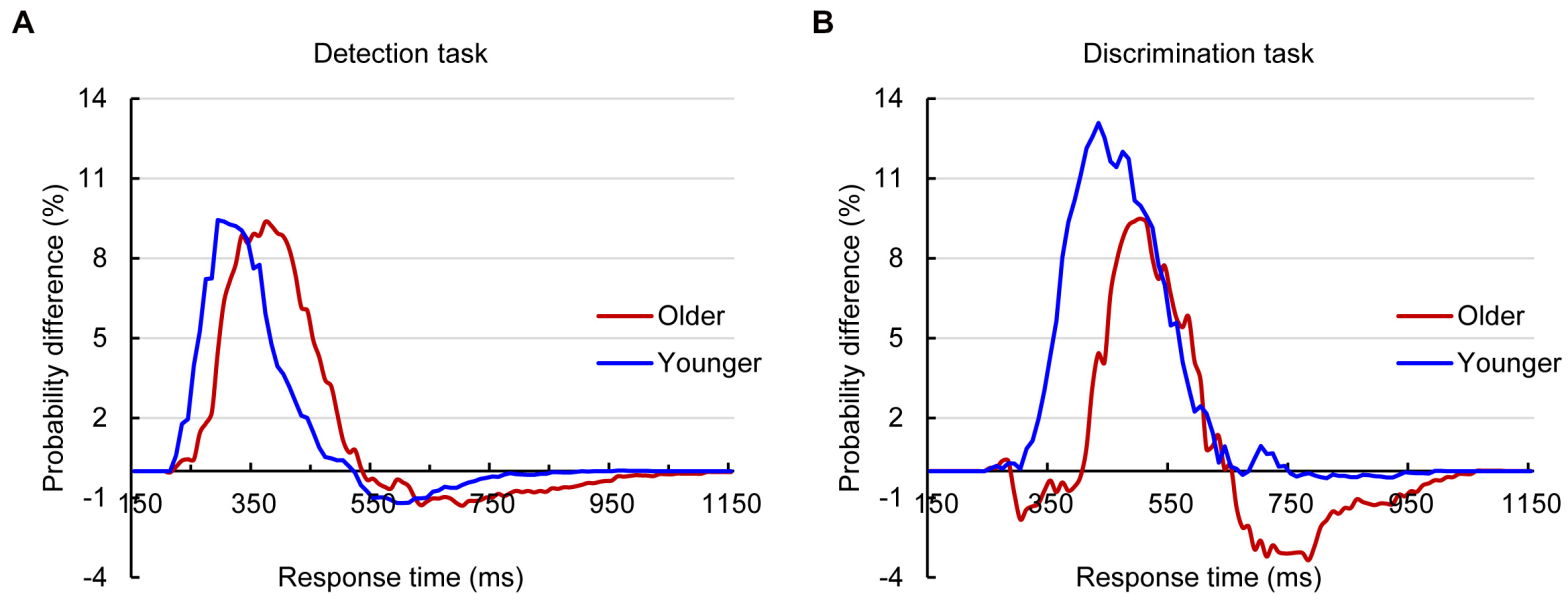
Probability difference between audiovisual CDFs and race model CDFs for older and younger adults in detection **(A)** and discrimination **(B)** tasks.

The analysis for RTs using the race model revealed significant AVI in both younger and older adults in the detection task and discrimination tasks (Figure 2). The independent *t* test revealed that the AVI of older adults was comparable to that of younger adults in the detection task (9.37% vs. 9.43%, *t_83_* = 0.698, *p* = 0.488, Figure 2A) but significantly lower than that of younger adults in the discrimination task (9.48% vs. 13.08%, *t_83_* = −2.952, *p* = 0.034, Figure 2B).

### 3.3. EEG data

#### 3.3.1. Detection task

**Figure 3:**
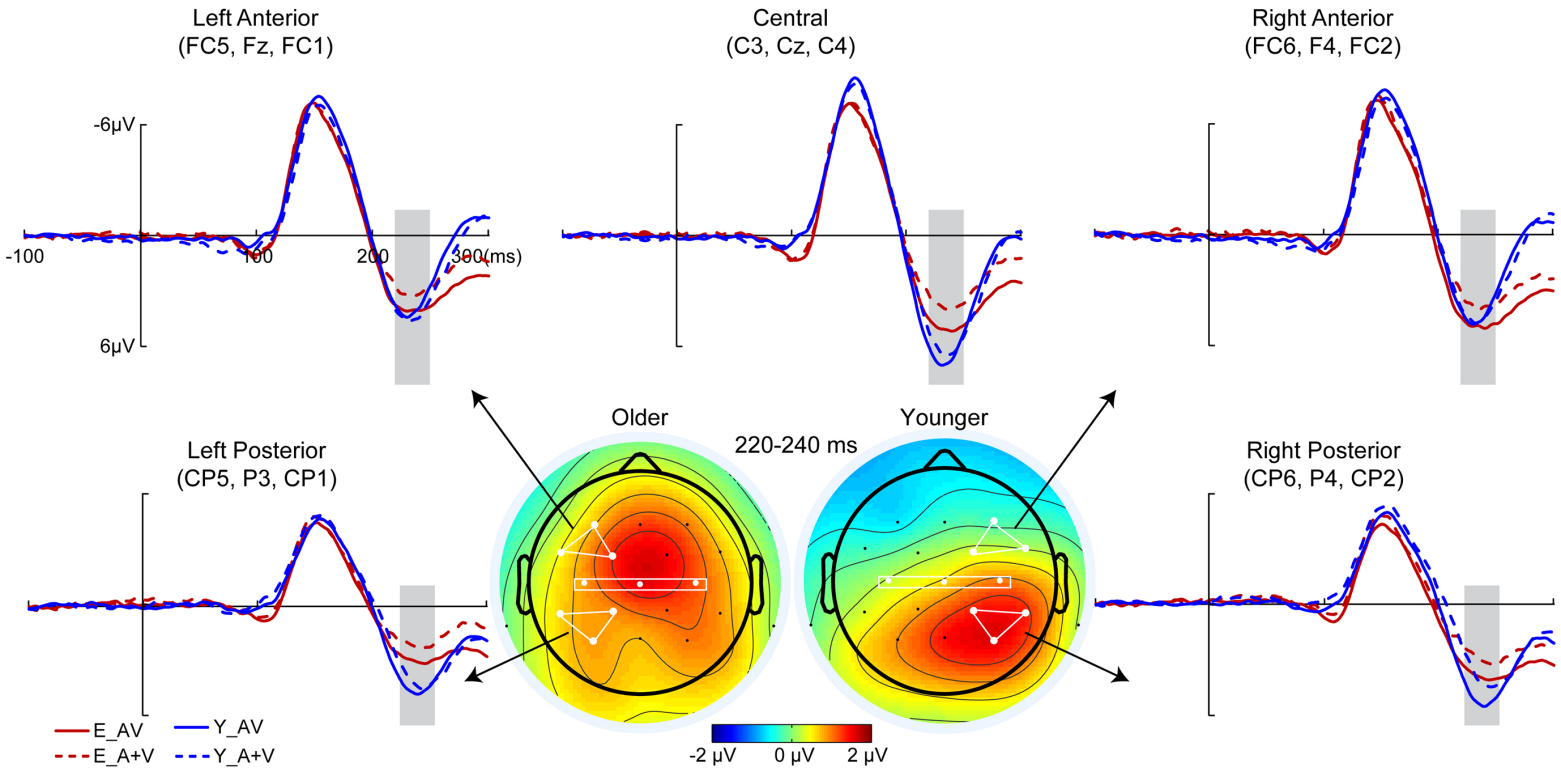
Grand-averaged event-related potentials and topography map of audiovisual integration for older and younger adults in the time window of 220–240 ms in the detection task. Grand-averaged event-related potentials of the left anterior are the mean amplitudes of FC5, F3, and FC1; right anterior are the mean amplitudes of FC6, F4, and FC2; central are the mean amplitudes of C3, Cz, and C4; left posterior are the mean amplitudes of CP5, P3, and CP1; and right posterior are the mean amplitudes of CP6, P4, and CP2. The time interval where audiovisual integration occurred is marked with gray squares in the ERP waves, and the darker the color (the larger the absolute value) on the topographic map, the stronger the audiovisual integration.

To remove the influence of action potential on ERP components, only a 400-ms time interval (100 ms prestimulus and 300 ms poststimulus points) was analyzed in the detection task. Pointwise running *t* tests revealed that significant AVI occurred at 220–240 ms (Figure 3). 2 (group: older, younger) × 5 (ROIs: left anterior, right anterior, central, left posterior, right posterior) ANOVA revealed a significant main effect of ROIs [*F*(4, 332) = 15.652, *p* < 0.001, η*_P_*^2^ = 0.133], indicating a higher amplitude in the right posterior than in the other ROIs. Additionally, there was a significant interaction between group and ROIs [*F*(4, 332) = 12.709, *p* < 0.001, η*_P_*^2^ = 0.159]. The *post hoc* analysis for group showed that for older adults, no significant difference was found between ROIs. However, for younger adults, the amplitude in the right posterior was significantly higher than the others (all *ps* < 0.001) and higher in the left posterior and central regions than in the right anterior and left anterior regions (all *ps* ≥ 0.152). There was no significant difference between the left posterior and central (*p* > 0.999), but there was a significantly higher amplitude in the right anterior than in the left anterior (*p* = 0.033). The *post hoc* analysis for ROIs found higher amplitude in the left anterior and right anterior (all *ps* ≤ 0.028) but lower amplitude in the right posterior (*p* = 0.038) for older adults than for younger adults; however, no significant difference was found between older and younger adults in the central (*p* = 0.205) and left posterior (*p* = 0.690). In addition, there was no significant main effect of group [*F*(1, 83) = 1.652, *p* = 0.202, η*_P_*^2^ = 0.020].

#### 3.3.2. Discrimination task

To further investigate whether the aging effect occurred in the late cognitive processing stage, a 700-ms time interval (100 ms prestimulus and 600 ms poststimulus points) was analyzed in the discrimination task. Pointwise running *t* tests revealed that significant AVI occurred at 220–240, 290–310, and 400–420 ms. In each AVI time interval, the mean amplitudes were submitted to 2 (group: older, younger) × 5 (ROIs: left anterior, right anterior, central, left posterior right posterior) ANOVA.

##### 3.3.2.1. 220–240 Ms

Similar to that in the detection task, there was a significant main effect of ROIs [*F*(4, 332) = 8.604, *p* < 0.001, η*_P_*^2^ = 0.094], indicating higher amplitude in the right posterior and central regions than in other ROIs (all *ps* ≤ 0.017). No significant main effect of group [*F*(1, 83) = 1.003, *p* = 0.319, η*_P_*^2^ = 0.012] or interaction between group and ROIs [*F*(4, 332) = 1.448, *p* = 0.234, η*_P_*^2^ = 0.017] was found.

##### 3.3.2.2. 290–310 Ms

There were significant main effects of group [*F*(1, 83) = 4.494, *p* = 0.037, η*_P_*^2^ = 0.051] and ROIs [*F*(4, 332) = 9.793, *p* < 0.001, η*_P_*^2^ = 0.106], indicating higher amplitudes for younger adults than for older adults in the left anterior, right anterior and central regions than in the left posterior and right posterior regions. Additionally, there was a significant interaction between group and ROIs [*F*(4, 332) = 3.957, *p* = 0.014, η*_P_*^2^ = 0.046]. The *post hoc* analysis for group showed that there was no significant difference between ROIs for older adults (all *ps* ≥ 0.324). Higher amplitude in left anterior and central than that in other ROIs (all *ps* ≤ 0.009) in right anterior and left posterior than that in right posterior (all *ps* ≤ 0.003) for younger adults; however, no significant difference was found between left anterior and central (all *ps* > 0.999) or between right anterior and left posterior (all *ps* > 0.999). The *post hoc* analysis for ROIs showed higher amplitudes for younger than for older adults in the left anterior (*p* = 0.014), central (*p* = 0.010) and right posterior (*p* = 0.009) but comparable amplitudes in the right anterior (*p* = 0.404) and right posterior (*p* = 0.412).

##### 3.3.2.3. 400–420 Ms

**Figure 4:**
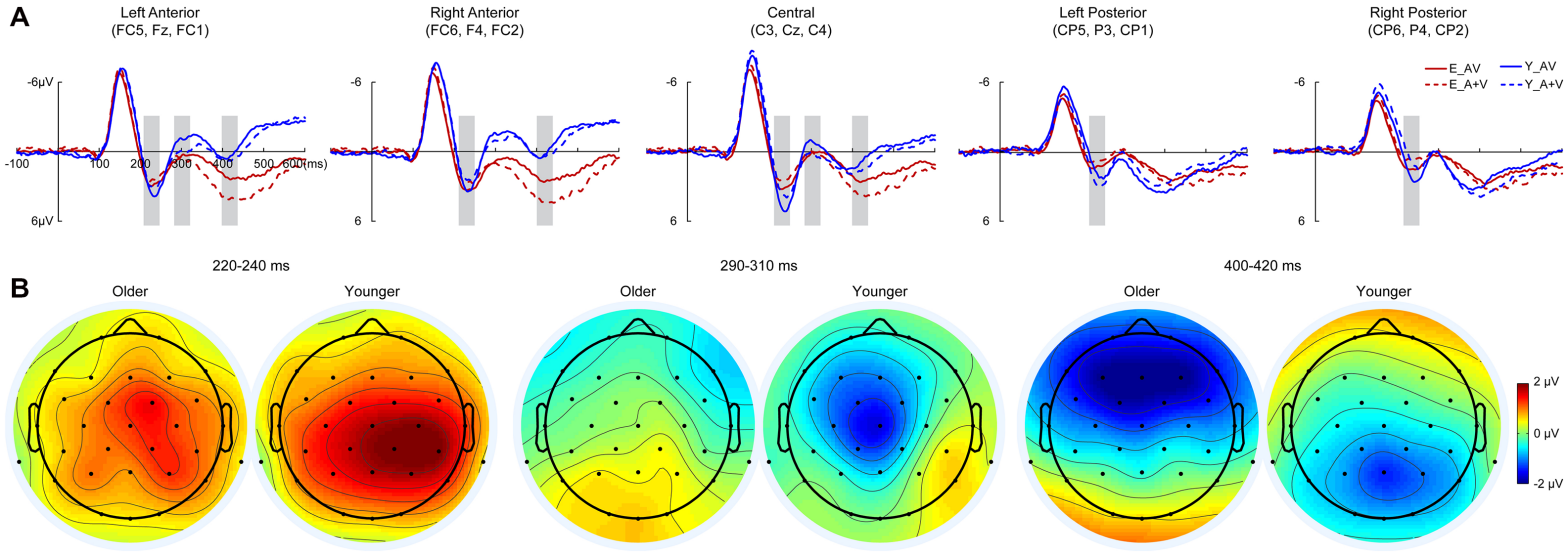
Grand-averaged event-related potentials **(A)** and topography map of audiovisual integration **(B)** for older and younger adults at intervals of 220–240, 290–310, and 420–440 ms in the discrimination task. Grand-averaged event-related potentials of the left anterior are the mean amplitudes of FC5, F3, and FC1; right anterior are the mean amplitudes of FC6, F4, and FC2; central are the mean amplitudes of C3, Cz, and C4; left posterior are the mean amplitudes of CP5, P3, and CP1; and right posterior are the mean amplitudes of CP6, P4, and CP2. The time interval where audiovisual integration occurred is marked with gray squares in the ERP waves, and the darker the color (the larger the absolute value) on the topographic map, the stronger the audiovisual integration.

There were no significant main effects of group [*F*(1, 83) = 2.260, *p* = 0.137, η*_P_*^2^ = 0.027] and ROIs [*F*(4, 332) = 2.854, *p* < 0.057, η*_P_*^2^ = 0.033]; however, the interaction between group and ROIs was significant [*F*(4, 332) = 25.879, *p* < 0.001, η*_P_*^2^ = 0.238]. The *post hoc* analysis showed that for the older group, there was a higher amplitude in the left anterior and right anterior than in other ROIs (all *ps* ≤ 0.030) in the central region than in the left posterior (*p* = 0.001) and right posterior (*p* < 0.001), but there was no significant difference between the left anterior and right anterior (*p* > 0.999) or between the left posterior and right posterior (*p* > 0.999). For younger adults, the amplitude was higher in the central, right posterior and right posterior than in the right anterior and left anterior (all *ps* ≤ 0.011); however, there was no significant difference among the central, right posterior and right posterior (all *ps* ≥ 0.208) or between the right anterior and left anterior (*p* > 0.999). The *post hoc* analysis for ROIs showed higher amplitudes in the left anterior (*p* = 0.001) and right anterior (*p* = 0.001) but lower amplitudes in the left posterior (*p* = 0.039) and right posterior (*p* = 0.026) for younger adults than for older adults, but there was no significant difference in centrality between older and younger adults (*p* = 0.236; Figure 4).

## 4. Discussion

The aim of the current study was to investigate when the aging effect occurred during auditory and visual merging processing and its neural mechanism using a detection task and discrimination task. The results found that during stimulus detection, no significant AVI difference was found between older and younger adults behaviorally; however, a higher AVI (220–240 ms) was found in the left posterior for younger adults, but there was no significant difference between brain regions for older adults. AVI was lower for older adults than for younger adults during stimulus discrimination, and attenuated AVI mainly occurred in the 290–310 ms time interval.

### 4.1. Comparable AVI in the stimulus detection stage

Inconsistent with Peiffer et al.’s study (Peiffer et al., 2007), our present study found that the AVI was comparable between older and younger adults in the detection task. There is general age-related slowing in uni-sensory and multisensory responses (Paige and Gutchess, 2017; Anderson, 2019; Jones and Noppeney, 2021), even in simple reaction times (Cliff et al., 2013); however, Peiffer et al. reported no significant difference in uni-sensory responses but a faster multisensory response for older adults than for younger adults. When the AVI was calculated, the appearance of enhanced AVI for older adults was observed, which might be an epiphenomenon and a unique report (Peiffer et al., 2007). Consistent with numerous previous studies, the response to uni-sensory and multisensory stimuli was slower for older adults than for younger adults in the current study (Laurienti et al., 2006; Grady, 2012; Diaconescu et al., 2013; Ren et al., 2020c), which further led to delayed AVI (Laurienti et al., 2006; Ren et al., 2020b, 2021). However, we first reported that the quantification of AVI was equivalent for the two age groups during simple meaningless stimulus detection.

In addition, consistent with the behavioral results, the current ERP analysis also showed no significant difference in the AVI amplitudes between older and younger adults at 220–240 ms, but further pairwise comparison showed no significant difference between ROIs for older adults but a higher AVI amplitude in the right posterior than the others for younger adults. With aging, brain structural and functional variables have been reported extensively, focusing on the core construct of compensatory scaffolding (Goh and Park, 2009; Reuter-Lorenz and Park, 2014). Studies have found that different from that for younger adults, the older adults recruited traditional unimodal information processing brain regions (Ren et al., 2018) and associated brain region (Diaconescu et al., 2013; Ren et al., 2020b) to process bimodal audiovisual information by reducing lateralization. Together with the behavioral and EEG results, we proposed that although there was no obvious diversity in behavioral expression, different neural representations occurred (Goh and Park, 2009; Reuter-Lorenz and Park, 2014), specifically reduced lateralization (Freiherr et al., 2013; Ren et al., 2020c) and shifted AVI regions (Davis et al., 2007; Ren et al., 2020a). However, considering the low spatial resolution of EEG studies, further fMRI studies are needed.

### 4.2. Lower AVI in the stimulus discrimination stage

Consistent with a previous study, the AVI was lower for older adults than for younger adults during the discrimination of meaningless auditory and visual stimuli (Wu et al., 2012; Ren et al., 2020b, 2021). The attention is a complex system in the brain that involves several different brain regions and mainly divided into three separate but interrelated networks: alerting, orienting, and executive control. Williams et al. (2016) investigated attention network using attention network test (ANT) while EEG recording, and found the older adults showed reduced alerting, but did not differ from younger adults in orienting or executive control (Ishigami et al., 2015; Williams et al., 2016). The AVI was higher in the attended condition than in the unattended condition (Talsma and Woldorff, 2005; Talsma et al., 2007, 2010; Tang et al., 2016); therefore, attention decline might be the most likely factor in the reduced AVI for older adults. Additionally, as in the stimulus detection task, there was no significant difference in the AVI amplitude between older and younger adults during 220–240 ms; however, AVI-related brain regions were different.

Furthermore, the AVI occurred for younger adults but was absent for older adults during 290–310 ms, and the AVI amplitude was higher in the left anterior and right anterior for older adults but in the central, right posterior and right posterior for younger adults during 400–420 ms. These results indicated that the attenuated AVI for older adults might be attributed to information processing in 290–310 ms, which mainly involved the N2 component. In discrimination tasks, the no-go N2 in the anterior was shown to reflect response inhibition (Folstein and Van Petten, 2008), and older adults have a inhibition deficit in go/no-go task (Rey-Mermet et al., 2018). Therefore, it is reasonable for a reduced AVI for older adults in the discrimination task, and we further proposed that the aging effect of AVI occurred as early as 220–240 ms, but the attenuated AVI mainly occurred in the later discriminating process at 290–310 ms.

In conclusion, there was a significant aging effect during AVI in multiple stages, but the older adults retained the ability to merge cross-modal information during the simple detection task attributed to the adaptive compensation mechanism. During stimulus discrimination, the AVI was attenuated, and it mainly occurred in the later discriminating stage at 290–310 ms, which was attributed to the attention suppression deficit.

## Data availability statement

The raw data supporting the conclusions of this article will be made available by the authors, without undue reservation.

## Ethics statement

The studies involving human participants were reviewed and approved by Second Affiliated Hospital of Guizhou University of Traditional Chinese Medicine. The patients/participants provided their written informed consent to participate in this study.

## Author contributions

YR and YL conceived and designed the experiments. RL, RQ, and JD collected the data. ZX and YR analyzed the data. YR wrote the draft manuscript with feedback from JY and WY. All authors contributed to the article and approved the submitted version.

## Funding

This study was partially supported by the Science and Technology Planning Project of Guizhou Province [QianKeHeJiChu-ZK (2021) General 120], the National Natural Science Foundation of China (32260198, 31800932, 31700973), and the Zhejiang Provincial Philosophy and Social Sciences Planning Project (22NDQN280YB).

## Conflict of interest

The authors declare that the research was conducted in the absence of any commercial or financial relationships that could be construed as potential conflicts of interest.

## Publisher’s note

All claims expressed in this article are solely those of the authors and do not necessarily represent those of their affiliated organizations, or those of the publisher, the editors and the reviewers. Any product that may be evaluated in this article, or claim that may be made by its manufacturer, is not guaranteed or endorsed by the publisher.

## Footnotes

^1^ http://sccn.ucsd.edu/eeglab/
